# Presynaptic Release-Regulating Alpha2 Autoreceptors: Potential Molecular Target for Ellagic Acid Nutraceutical Properties

**DOI:** 10.3390/antiox10111759

**Published:** 2021-11-04

**Authors:** Isabella Romeo, Giulia Vallarino, Federica Turrini, Alessandra Roggeri, Guendalina Olivero, Raffaella Boggia, Stefano Alcaro, Giosuè Costa, Anna Pittaluga

**Affiliations:** 1 Net4Science Academic Spin-Off, Università degli Studi “Magna Græcia” di Catanzaro, Campus “S. Venuta”, Viale Europa, 88100 Catanzaro, Italy; alcaro@unicz.it; 2 Dipartimento di Scienze della Salute, Università degli Studi “Magna Græcia” di Catanzaro, Campus “S. Venuta”, Viale Europa, 88100 Catanzaro, Italy; 3 Associazione CRISEA—Centro di Ricerca e Servizi Avanzati per l’Innovazione Rurale, Località Condoleo, 88055 Belcastro, Italy; 4 Department of Pharmacy, University of Genoa, Viale Cembrano, 4, 16148 Genoa, Italy; giulia.vallarino94@gmail.com (G.V.); turrini@difar.unige.it (F.T.); roggeri@difar.unige.it (A.R.); olivero@difar.unige.it (G.O.); boggia@difar.unige.it (R.B.); pittalug@difar.unige.it (A.P.); 5 IRCCS Ospedale Policlinico San Martino, 16145 Genova, Italy

**Keywords:** pomegranate tannins, ellagic acid, molecular modelling, α_2_-adrenoreceptors, α_2_-ARs, molecular dynamic simulations, antioxidant, natural compounds, antidepressant activity, food chemistry

## Abstract

Polyphenol ellagic acid (EA) possesses antioxidant, anti-inflammatory, anti-carcinogenic, anti-diabetic and cardio protection activities, making it an interesting multi-targeting profile. EA also controls the central nervous system (CNS), since it was proven to reduce the immobility time of mice in both the forced swimming and the tail-suspension tests, with an efficiency comparable to that of classic antidepressants. Interestingly, the anti-depressant-like effect was almost nulled by the concomitant administration of selective antagonists of the noradrenergic receptors, suggesting the involvement of these cellular targets in the central effects elicited by EA and its derivatives. By in silico and in vitro studies, we discuss how EA engages with human α_2A_-ARs and α_2C_-AR catalytic pockets, comparing EA behaviour with that of known agonists and antagonists. Structurally, the hydrophobic residues surrounding the α_2A_-AR pocket confer specificity on the intermolecular interactions and hence lead to favourable binding of EA in the α_2A_-AR, with respect to α_2C_-AR. Moreover, EA seems to better accommodate within α_2A_-ARs into the TM5 area, close to S200 and S204, which play a crucial role for activation of aminergic GPCRs such as the α_2_-AR, highlighting its promising role as a partial agonist. Consistently, EA mimics clonidine in inhibiting noradrenaline exocytosis from hippocampal nerve endings in a yohimbine-sensitive fashion that confirms the engagement of naïve α_2_-ARs in the EA-mediated effect.

## 1. Introduction

Pomegranate (*Punica granatum* L.) is a very ancient edible fruit originating in the Middle East and North Africa, used from the dawn of history as a healing and health-promoting fruit in traditional medicine [1]. In the past decades, this rustic crop has obtained high popularity as a nutraceuticals source, becoming a high-value crop. Moreover, it has demonstrated increased importance due to its adaptability to different climatic conditions, its resilience and longevity, and its high drought and salinity resistance [2].

Today, pomegranate cultivation is widely spread in many tropical and subtropical regions, and about two million tons of fruits are produced annually worldwide [3]. Particularly, India, Iran, China, Turkey, the United States, Spain, South Africa, Peru, Chile, and Argentina represent the major producers and exporters of this fruit [4].

Pomegranate fruit is of great economic and nutritional interest, and it is in high demand due to its wide range of industrial uses, especially for direct consumption, for juice production, and oil extraction from its seeds [2]. The nutritional value of pomegranate is linked to its naturally high content of phenolic compounds with antioxidant properties [5]. Many studies, mainly in vitro, demonstrated the health benefits and the functional properties of this fruit in the prevention of several peripheral disorders such as cancer, cardiovascular diseases, chronic inflammatory diseases, and metabolic disorders (i.e., diabetes, obesity) [6]. Moreover, the beneficial effects of pomegranate phenolic compounds on central neuroinflammatory and neurodegenerative pathologies, including multiple sclerosis, Alzheimer’s disease, Parkinson’s diseases, epilepsy, and depression, have been highlighted too [7,8,9].

The main industrial product from pomegranate is its juice, obtained by aril squeezing, which represents the edible portion of the fruit, but recently there has been an increased focus on pomegranate by-products as a source of nutraceuticals. It has been determined that by-products, especially the peels, have higher levels of bioactive compounds and antioxidant activity than juice, opening new possibilities for pomegranate manufacturers to recover and exploit these by-products in a zero-waste economy perspective [10]. A major class of compounds in pomegranate fruit is the hydrolysable tannins, including ellagic acid, punicalagin, and gallic acid [11].

**Figure 1:**
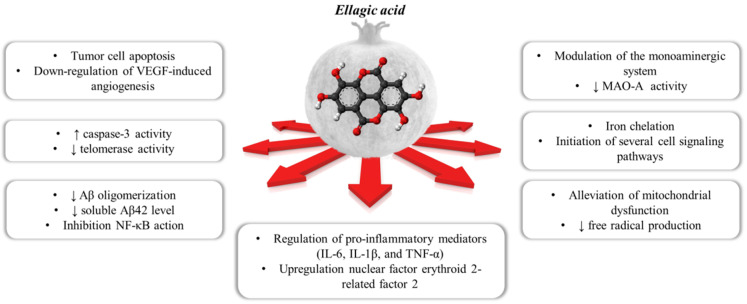
The polypharmacological effects regulated by ellagic acid contained within the pomegranate.

Ellagic acid (EA) is a chromene-dione derivative (3,7,8-tetrahydroxy-chromeno[5,4,3-cde]chromene-5,10-dione), which is derived from the spontaneously lactonization of hexahydroxydyphenic acid (HHDP) [12]. In its free form, or as constituent of ellagitannins (ETs), or conjugates with different monosaccharides (EA-glycosides), EA is considered the main phenolic compound responsible for the numerous health properties of pomegranate [12]. ETs are esters of gallic acid (GA) and hexahydroxydiphenic acid (HHDP) units, connected with mainly β-d-glucose as sugar residue. Punicalagin (2,3-(S)-hexahydroxydiphenoyl-4,6-(S,S)-gallagyl-d-glucose), a large molecule consisting of ellagic acid and gallagic acid linked via a glucose unit, is the most abundant ET in pomegranate and it is specific to the *Punica* genus [8]. Data from preclinical studies in the literature support the healthy properties of both EA and ETs. These include the ability to interfere with tumor cell proliferation, the cell cycle, invasion and angiogenesis by making it a multi-target candidate for various cancer treatments [13]. It also places a particular emphasis on the role of EA in central inflammatory and (auto)-immunological diseases, but also for depression, anxiety and aged-related neurological impairments [7,14,15,16] (Figure 1).

As far as the mood disorders are concerned, EA was proven to reduce the immobility time of mice in both the forced swimming and the tail-suspension tests, with an efficiency comparable to that of classic antidepressants [17]. Interestingly, the anti-depressant-like activity was almost nulled by the concomitant administration of selective antagonists of the noradrenergic receptors (namely the α_1_, the α_2_ and the β receptors) and by modulators of the serotonergic systems as well (including receptor antagonists and synthesis inhibitors) [17,18], suggesting the involvement of these cellular targets in the central effects elicited by EA and its derivatives.

Among the noradrenergic receptors, we focussed on the α_2_ receptors (α_2_-ARs) that, in the central nervous system (CNS), act as presynaptic inhibitory autoreceptors in noradrenergic nerve endings/varicosities [19,20]. The α_2_-ARs are indirectly tuned by antidepressants acting as noradrenaline (NA) re-uptake inhibitors (NRI) [21]. By increasing NA bioavailability in the synaptic cleft, these drugs cause the continuous stimulation of the presynaptic α_2_-Ars, leading to their down regulation. A comparable outcome also can be triggered by the continuous direct activation of the presynaptic α_2_-ARs with agonists. In both cases, the final outcome is the silencing of the presynaptic mechanism of autocontrol of the release of the biogenic amine and, consequently, the reinforcement of the noradrenergic transmission [22,23,24]. Notably, the α_2_-ARs also exist in astrocytes, where they control the phenotype of the glial cells favouring the non-inflammatory one [25]; whether and how these receptors desensitize was so far scarcely investigated.

The α_2_-ARs are G protein coupled receptors (GPCRs) negatively associated to the adenylyl cyclase (AC) that reduce the gating of the voltage-operated calcium channels (VOCCs), concomitantly favouring the opening of the K^+^-channels, to inhibit cellular functions. There are three characterized α_2_-AR subtypes, the α_2A_-AR_,_ α_2B_-AR and α_2C_-AR [26], which are well conserved across mammals and are differently distributed in postganglionic sympathetic neurons and CNS noradrenergic neurons.

The α_2A_-ARs and the α_2C_-ARs are mainly expressed in noradrenergic neuronal projections from the Locus coeruleus to other central regions, with higher expression on the varicosities in dendritic and axonal processes, as well as in nerve terminals [20]. The α_2A_-ARs and the α_2C_-ARs are also present peripherally, on postganglionic sympathetic neurons, where again they act as inhibitory autoreceptors. Differently, the α_2B_-ARs are preferentially expressed in the periphery, their presence in the CNS still representing a matter of debate [27].

We recently demonstrated that the systemic administration of an orally available formulation of EA efficiently recovers the NA exocytosis in the cortex of aged mice, restoring it to a level comparable to that observed in young animals [8,9,28], and also reducing the endogenous levels of proinflammatory cytokines [28]. Taking into consideration that (i) the production of pro-inflammatory cytokines is tuned by NA [29,30,31,32], (ii) the efficiency of NA transmission is modulated by cytokines [22,33], and (iii) a common trait of the cytokines-NA cross-talk in the CNS is represented by the noradrenergic α_2_-ARs, we hypothesized that these receptors could be involved in the beneficial effects exerted by EA in aged mice.

To test our hypothesis, we focused on the α_2A_-ARs and the α_2C_-ARs, because of their preferential central distribution that would support their involvement in the control of mood disorders and in the anti-depressant activity. We carried out in-silico studies to explore the binding modes of EA into human α_2A_-ARs and α_2C_-AR catalytic pockets, comparing EA behaviour with those ones of a known agonist (clonidine) or antagonist (yohimbine), respectively. Through molecular docking and molecular dynamic simulations (MDs), we investigated whether the absence of the positively charged center in the EA could affect the binding to the α_2A_-ARs structures. Then, we applied a functional in vitro experimental approach to evaluate whether and how EA interacts with naïve α_2_-ARs.

## 2. Materials and Methods

### 2.1. Computational Protocols

All computational studies were carried out by means of Schrödinger Suite 2018-1 [34]. The x-ray crystallographic structures of the α_2A_-AR in complex with a partial agonist (PDB code: 6KUY) [26,35], and with the antagonist (PDB code: 6KUX) [36], and the α_2C_-AR in complex with an antagonist (PDB code: 6KUW) [37] were used. α_2A_-AR and α_2C_-AR structures were prepared by using the Maestro Protein Preparation Wizard [38] tool. All the hydrogen atoms were added, and the bond orders and the formal charges were adjusted for the hetero groups. The protonation states for all amino acids at physiological pH were calculated according to the Epik tool [39], and in particular, all aspartate residues were deprotonated in the inactive state of GPCRs, except for D113 which was protonated in the intermediate state. Missing residue atoms and loops were filled. Water molecules farther than 5 Å from heteroatoms were deleted. The structures were refined to optimize hydrogen-bonds and energy minimized by using OPLS_2005 as a force field at pH 7.4 [40,41]. For the ligands preparation, three-dimensional (3D) coordinates were generated for the ellagic acid, the partial agonist, and the antagonist such as clonidine and yohimbine, respectively, with LigPrep [42]. Then, target binding sites were defined by means of a regular grid box of about 27,000 Å^3^ centred on the co-crystallized ligands for each structure. All docking simulations were computed using the Glide [43] ligand flexible algorithm, at the standard-precision (SP) level. The best docked poses of ellagic acid, yohimbine and clonidine in complex to α_2A_-AR and α_2C_-AR structures were submitted to 50 ns of molecular dynamics simulations (MDs) by using Desmond ver. 4.2 [44]. The POPC membrane was set up on the retrieved membrane coordinates by using the Orientation of Proteins in Membranes (OPM) database [45] for each PDB structure. The systems were solvated in the TIP3P explicit solvent model and counter ions were added to neutralize the system net charge. The Desmond membrane relaxation protocol was used to equilibrate each system [46]. After optimization of the solvated models, the default equilibration protocol of Desmond was used to relax the whole systems which included two energy minimizations of 2000 steps: in the first run, the systems were restrained with a force constant of 50 kcal mol^−1^ A^−1^, while in the second one, the whole systems were released without any restrains. The final MD production for the three complexes was carried out for a simulation time of 50 ns. The following conditions for MDs were used: NPT ensemble, a temperature of 300 K, a pressure of 1 bar, with the Berendsen thermostat-barostat, a recording interval equal to 250 ps both for energy and for trajectory collecting 1000 frames for each simulation. In order to examine the ligand stability in the respective accommodation into the ARs, the distance between the centroid of the ligand and D113, S200 and S204 residues for α_2A_-ARs, and D131, S214 and S218 residues for α_2C_-ARs, were calculated [47]. The subsequent free energy calculation for each complex was calculated. One thousand snapshots from 50 ns of MDs were applied for the MM/GBSA free energy calculations [48,49] based on the following equation:

(1) $$\Delta G_{bind}=G_{comp}-\;G_{pro}-G_{lig}={\Delta E}_{ele}+{\Delta E}_{vdw}+{\Delta E}_{GB}+{\Delta E}_{surf}$$

where G*_comp_*, G*_pro_* and G*_lig_* denotes the free energy of the complex, protein and the ligand; by splitting the energy contribution, it referred to ΔE*_ele_* and ΔE*_vdw_* as the gas-phase interaction energy between protein and ligand, thus including the electrostatic energy term and van der Waals energy term, respectively. Meanwhile, ΔE*_GB_* and ΔE*_surf_* indicate the polar and nonpolar desolvation free energy, respectively. The implicit solvation was calculated using the GB model [50], while the non-polar solvation energy was calculated using the solvent accessible surface area algorithm.

ΔG*_bind_* reported in this study omitted the entropy contribution due to its relatively high computational demand and the lake of information of the conformational entropy that could lead to the introduction of additional error into the results [51].

### 2.2. In Vitro Functional Pharmacological Studies: Animals

Mice (male, strain C57BL/6J) were obtained from Charles River (Calco, Italy) and housed in the animal facility of the Department of Pharmacy (DIFAR), Pharmacology and Toxicology Section (Genoa, Italy), under controlled environmental conditions (ambient temperature = 22 °C, humidity = 40%) on a 12-h light/dark cycle with freely available food and water. Mice were euthanized by cervical dislocation, followed by decapitation, and their hippocampi rapidly removed.

Animal care and experimental procedures were in accordance with the ARRIVE guidelines and the European Communities Parliament and Council Directive of 22 September 2010 (2010/63/EU), and with the Italian D.L. n. 26/2014, and were approved by the Local Committee for Animal Care and Welfare of the University of Genova and the Italian Ministry of Health (DDL 26/2014 and previous legislation; protocol number n° 75F11.N.IMY). In line with the 3Rs principles (reduction, refinement and replacement), all efforts were made to minimize the number of animals used and their suffering.

### 2.3. Preparation of Synaptosomes

Synaptosomes are pinched-off nerve terminals that contain structures (e.g., vesicles, mitochondria, endoplasmic reticulum, synthetic and enzymatic pathways, see Olivero et al., 2021 [52]) that are present in the neuronal processes they originate from, confirming their presynaptic origins. Synaptosomes can take up, synthesize, metabolize, store, and release transmitters, and possess naïve receptors and transporters, whose activation controls the synaptosomal functions, particularly transmitter exocytosis. Synaptosomes are obtained by homogenizing brain tissues, which are then isolated by density-gradient centrifugation as previously described [53]. Briefly, the tissue was homogenized in 10 volumes of 0.32 M sucrose, buffered to pH 7.4 with Tris-(hydroxymethyl)-amino methane (TRIS, final concentration 0.01 M) using a glass/Teflon tissue grinder (clearance 0.25 mm). The homogenate was centrifuged at 1000× *g* for 5 min to remove nuclei and debris; the supernatant was gently layered on a discontinuous Percoll gradient (6, 10, and 20% *v*/*v* in Tris-buffered 0.32 M sucrose) and then centrifuged at 33,500× *g* for 6 min. The layer between 10 and 20% Percoll, which correspond to the synaptosomal fraction, was collected, and washed by centrifugation at 19,000× *g* for 15 min. The synaptosomal pellet was then resuspended in a physiological medium of the following composition (mM): NaCl, 140; KCl, 3; MgSO_4_, 1.2; CaCl_2_, 1.2; NaH_2_PO_4_, 1.2; NaHCO_3_, 5; HEPES, 10; glucose, 10; pH 7.4.

### 2.4. Release Experiments

**Figure 2:**
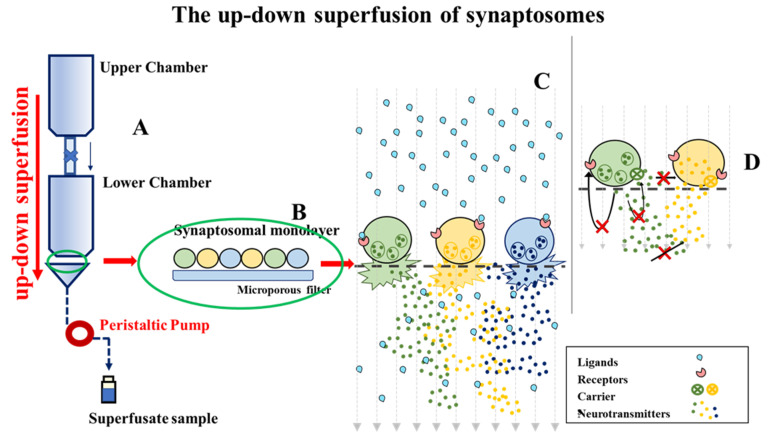
The superfusion apparatus (see text) [54,55].

Hippocampal synaptosomes were incubated for 15 min at 37 °C in a rotary water bath with the radioactive tracer [^3^H]NA (f.c.: 30 nM), in the presence of 0.1 µM 6-nitroquipazine to avoid the false labelling of serotonergic terminals. Identical aliquots of the synaptosomal suspension were then stratified as a thin monolayer on microporous filters at the bottom of parallel chambers maintained at 37 °C of a Superfusion System (Ugo Basile, Comerio, Varese, Italy) [54,55], as first proposed by Raiteri and colleagues in 1974 (Figure 2) [54,55,56].

The superfusion apparatus consists of 20 units of superfusion, composed of an upper chamber (to heat the superfusion medium) and a lower chamber, where synaptosomes are stratified on a microporous filter as a monolayer (**A**). The superfusion medium is continuously collected by means of a peristaltic pump at a constant flow rate (0.5 mL/L). This continuous up-down superfusion of the synaptosomal monolayer (**B**) assures the removal of any endogenous substances (**C**), therefore minimizing any retrograde effects (**D**). The presynaptic naïve receptors on the superfused synaptosomes are therefore “ligand-free”, and their release-regulating activity can be triggered only by adding exogenous agonist(s) to the superfusion medium to activate the receptor-mediated signal (**D**). Differently, in this experimental paradigm, antagonists are “*per se*” inactive but became functionally active when added concomitantly to agonists, because of their ability to compete with agonists at presynaptic receptors modulating the transmitter exocytosis. The selective labelling with radioactive tracers (i.e., [^3^H]NA) of a specific subpopulation of terminals allows monitoring of the effects elicited by the receptors located on a selected subpopulation of nerve terminals (in our case the noradrenergic ones), impeding artefacts that may originate from the presence of the receptor under study on other subfamilies of terminals. The quantification of the radioactive tracer in the collected samples permits the correlation of the changes in transmitter exocytosis to the concentrations of the ligands in the superfusion medium and, therefore, to the activation of the presynaptic receptors controlling the efficiency of transmitter release (elicited in our case by a depolarizing stimulus, i.e., the 12 mM KCl **C**).

Synaptosomes were continuously “up-down” superfused with an isosmotic-isotonic ionic solution at a constant flow rate (0.5 mL/min) for 36 min to equilibrate the system. At t = 39, synaptosomes were transiently exposed (90 s) to a high KCl-containing medium (12 mM, KCl, substituting for an equimolar concentration of NaCl), in the absence or in the presence of clonidine and ellagic acid, alone or concomitantly added with yohimbine. Superfusate fractions were collected as follows: two 3-min fractions (basal release), one before (t = 35–39 min, b1) and one after (t = 45–48 min, b3) a 6-min fraction (t = 39–45 min, evoked release, b2). The 12 mM KCl-evoked [^3^H]NA overflow was calculated by subtracting the neurotransmitter content in the b1 and b3 fractions from that in the b2 fraction. The amount of radioactivity in each superfusate fraction was expressed as a percentage of the total synaptosomal radioactivity. Data are reported as the mean ± SEM of independent determinations obtained in different experiments run in triplicate (at least three superfusion chambers for each experimental condition). The effects of EA, yohimbine and clonidine were expressed as a percentage of the KCl-induced overflow in the absence of these ligands (percent of residual).

### 2.5. Western Blot Analysis

Mouse hippocampus was homogenated in RIPA lysis buffer (10 mM Tris, pH 7.4, 150 mM NaCl, 1 mM EDTA, 0.1% SDS, 1% Triton X-100, 1 mM sodium orthovanadate, and protease inhibitors) as previously described [57]. Proteins were quantified with a BCA assay, separated by SDS-10% PAGE (µg/lane as indicated in the figures) and blotted onto PVDF membranes. Membranes were probed with rabbit α_2A_ adrenergic receptor polyclonal antibody (1:250, PA1-048, Invitrogen, Thermo Fisher Scientific, Waltham, MA, USA) and with mouse α_2C_ adrenergic receptor monoclonal antibody (1:500, S330A-80, Invitrogen, Thermo Fisher Scientific) overnight at 4 °C. Membranes were then incubated for 1 h at room temperature with the appropriate horseradish peroxidase-linked secondary antibodies (1:5000, A9044 and A9169, Sigma-Aldrich, St. Louis, MO, USA). Immunoblots were visualized using an ECL (enhanced chemiluminescence) Western blotting detection system. Images were acquired using the Alliance LD6 images capture system (Uvitec, Cambridge, UK) and analysed by UVI-1D software (Uvitec, Cambridge, UK).

### 2.6. Statistical Analysis

For data handling/statistics and graph drawing, the SigmaPlot 10 data analysis and graphing software package was used. Multiple comparisons were performed with analysis of variance (ANOVA) followed by Tukey’s multiple-comparisons test. Data were considered significant for *p* < 0.05 at least.

### 2.7. Chemicals

Noradrenaline, levo [7-^3^H] (specific activity 12.1 Ci/mmol), was from Perkin Elmer (Boston, MA, USA). Ellagic acid, yohimbine, clonidine and 6-nitroquipazine were from Sigma Aldrich. Western blotting detection system Immobilon Forte Western HRP substrate was from Merck (Darmstad, Germany).

## 3. Results and Discussion

### 3.1. Computational Studies

In silico studies were carried out to explore the binding modes of EA into α_2A_-ARs and α_2C_-AR catalytic pockets. In addition to this, these specific docking studies were carried out to investigate the possible differences and similarities of the interactions established between a known agonist (clonidine) or antagonist (yohimbine) with the receptors. For the current analysis, the crystal structures of the human α_2A_-AR in complex with the indole derivative, a partial agonist (PDB code: 6KUY) [36], α_2A_-AR in complex with the naphthyridine derivative, an antagonist (PDB code: 6KUX) [58], and, α_2C_-AR in complex with the naphthyridine derivative, an antagonist (PDB code: 6KUW [37], were used.

#### 3.1.1. Molecular Docking Studies

**Figure 3:**
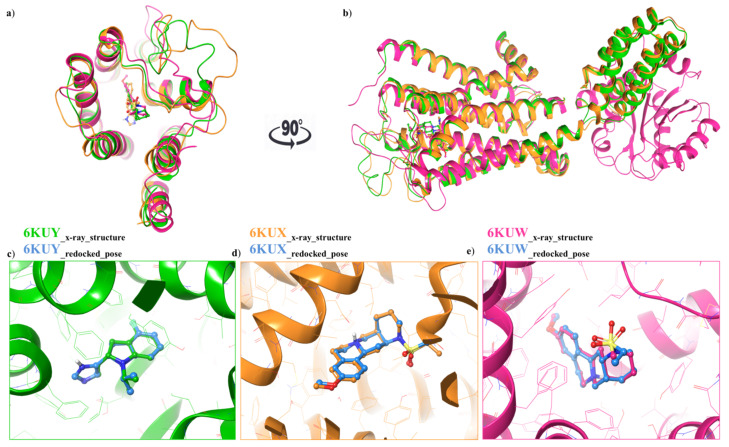
(**a**) Top view: Ribbon representation of overlaid binding orientation of co-crystallized ligands into the binding site of α_2A_-AR_6KUY_ (orange), α_2A_-AR_6KUX_ (green) and α_2C_-AR_6KUW_ (magenta) structures; (**b**) Front view of the superposed α_2A-C_-ARSs. Three-dimensional superimposition between the re-docked pose (blue carbon ball and sticks) and the conformation of the native ligand; (**c**) Indole derivative into the binding site of 6KUY; (**d**) Naphthyridine derivative into both 6KUX (green) and (**e**) 6KUW (magenta) X-ray structures.

For each structure, the docking protocol was validated by docking the co-crystallized ligand into the binding site (Figure 3a,b). Root mean square deviation (RMSD) values between the native pose of α_2A_-AR_6KUY_, α_2A_-AR_6KUX_ and α_2C_-AR6_KUW_ ligands and the related best re-docked conformations were found to be 0.19 Å (Figure 3c), 0.89 Å (Figure 3d), and 0.39 Å (Figure 3e), respectively, thus revealing the reliability of docking protocol (Figure 3a–c).

**Figure 4:**
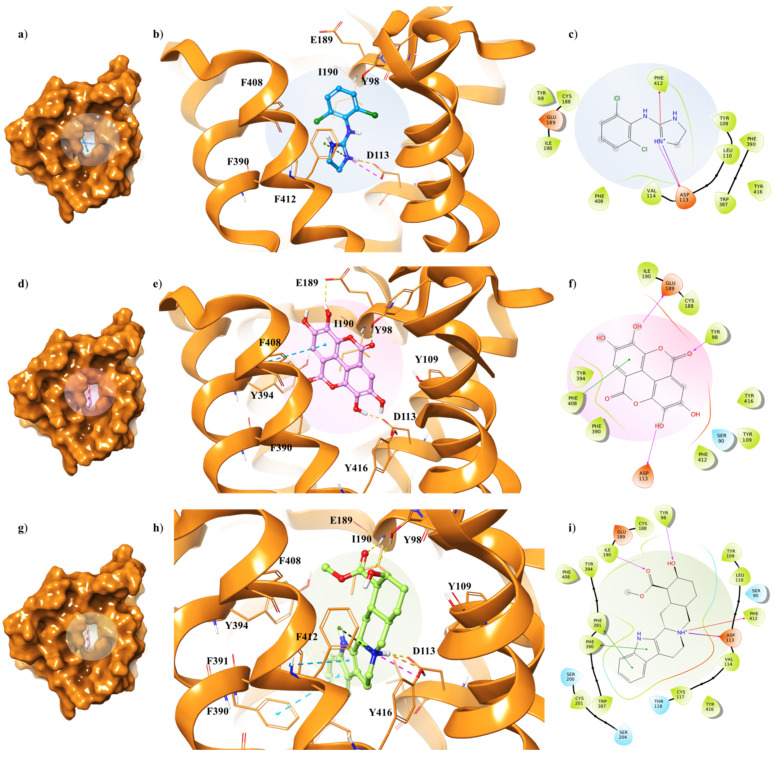
Key contacting elements inside (**a**–**c**) the α_2A_-AR_6KUX_/clonidine, (**d**–**f**) the α_2A_-AR_6KUX_/EA, and (**g**–**i**) the α_2A_-AR_6KUX_/yohimbine best docked pose. Panels (**b,e**,**h**) show all side chains involved in H-bonds (violet), π-π interactions (cyan) and π-cation interactions (red) in stick representation. Panels (**a**,**d**,**g**) show the surface area of α_2A_-AR_6KUX_ complexed to clonidine, EA and yohimbine, respectively. The surface area of the receptor is shown in solid orange solid. 2D representation of the key interactions of (**c**) clonidine, (**f**) EA and (**i**) yohimbine into the α_2A_-AR_6KUY_ structure.

The three ligands were docked to the minimized structures of the α_2_-ARs subtypes A and C, respectively (Table 1). Regarding yohimbine in complex to α_2A_-AR_6KUX_, it was observed that it was able to better recognize the orthosteric pocket of the receptor, compared to EA and clonidine. In detail, the positively charged amine group in the pyrido[1,2-b]isoquinoline displays a hydrogen bond with D113 (1.81 Å) and a π-cation interaction with F412. The hydroxyl and the carboxylic group create a hydrogen bond with Y98 side chain and I190 backbone, respectively. Furthermore, the indole moiety enhances the binding by two π-π interactions with F390 and F391 (Figure 4g–i).

The peculiar structure of the EA is buried in a hydrophobic core formed by Y98, Y109, I190, F390, Y394, F408, F412 and Y416 residues (Figure 4f). The receptor–ligand interaction is then stabilized by H-bond interactions between the hydroxyl groups of the two gallic acid motifs and the side chains of D113 and E189, which lie on the extracellular loop 2 (XL2), directly linked to the TM5 [35]. Furthermore, a π-π interaction occurs in proximity with the receptor exterior, between F408 and one of the aryl rings; meanwhile, the oxygen of the carbonyl group of the EA forms the hydrogen bond with the hydroxyl group of Y98 (Figure 4d,e).

Clonidine docking pose shows a very similar orientation to that of the EA into the α_2A_-AR_6KUX_ binding cavity, thus pointing the positive charge of the imidazole ring towards D113 and F412, through a H-bond and a π-cation interaction, respectively, acting as an anchor within the binding site (Figure 4a–c).

**Figure 5:**
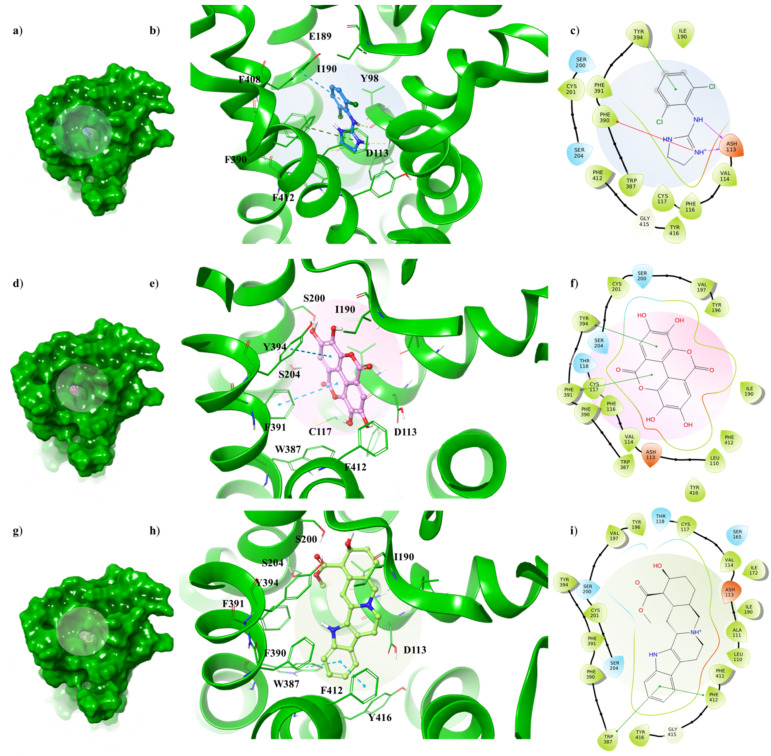
Key contacting elements inside (**a**–**c**) the α_2A_-AR_6KUY_/clonidine, (**d**–**f**) the α_2A_-AR_6KUY_/EA, and (**g**–**i**) the α_2A_-AR_6KUY_/yohimbine best docked pose. Panels (**b**,**e**,**h**) show all side chains involved in H-bonds (violet), π-π interactions (cyan) and π-cation interactions (red) in stick representation. Panels (**a**,**d,g**) show the surface area of α_2A_-AR_6KUY_ complexed to clonidine, EA and yohimbine, respectively. The surface area of the receptor is shown in solid green. 2D representation of the key interactions of (**c**) clonidine, (**f**) EA and (**i**) yohimbine into the α_2A_-AR_6KUY_ structure.

Although α_2A_-AR_6KUX_ and α_2A_-AR_6KUY_ are characterized by a different rearrangement of the side chains of the hydrophobic residues into the binding pocket, the indole moiety of the yohimbine into α_2A_-AR_6KUY_ can create a π-π interaction with F412 and W438 (Figure 5g–i). Instead, EA interacts with F391 and Y394 by means of one gallic acid motif (Figure 5d–f). When clonidine is docked to α_2A_-AR_6KUY_, the 2-aminoimidazoline group forms two hydrogen bonds with D113, and a π-cation interaction with F390, while the dichlorophenyl ring stretches towards the extracellular solvent to bind the Y394 residue (Figure 5a–c).

**Figure 6:**
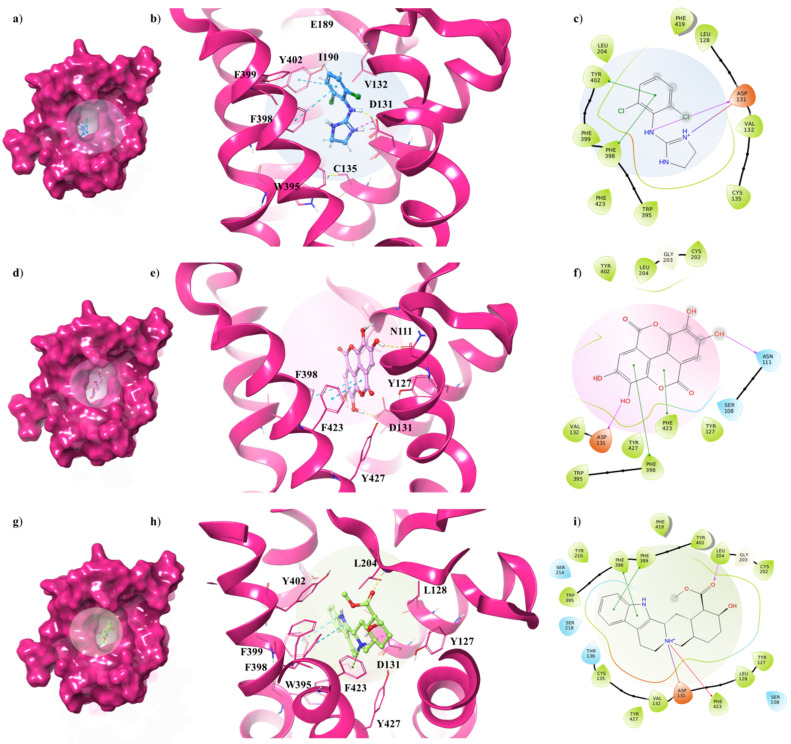
Key contacting elements inside (**a**–**c**) the α_2A_-AR_6KUW_/clonidine, (**d**–**f**) the α_2A_-AR_6KUW_/EA, and (**g**–**i**) the α_2_A-AR_6KUW_/yohimbine best docked pose. Panels (**b**,**e**,**h**) show all side chains involved in H-bonds (violet), π-π interactions (cyan) and π-cation interactions (red) in stick representation. Panels (**a**,**d**,**g**) show the surface area of α_2A_-AR_6KUW_ complexed to clonidine, EA and yohimbine, respectively. The surface area of the receptor is shown in solid magenta. 2D representation of the key interactions of (**c**) clonidine, (**f**) EA and (**i**) yohimbine into α_2A_-AR_6KUY_ structure.

Concerning the α_2C_-AR_6KUW_ subtype, docking analysis highlights that yohimbine forms a conserved salt bridge with the carboxylate of D131 and, also, a π-cation interaction with F423 and F391 (Figure 6a–c).

The antagonist is surrounded by nine aromatic residues (Y127, Y210, W395, F398, F399, Y402, F419, F423, Y427) on TM6 and TM7, and three other residues (L128, V132, L204) forming the hydrophobic environment of the binding cavity. The indole moiety interacts with F398 and F399 by means of two π-π interactions, while the carboxyl group engages a H-bond with L204, one of the pivotal residues in establishing subtype selectivity [47].

EA is well accommodated into the α_2C_-AR pocket and interacts with the same residues with which yohimbine interacts, except that one hydroxyl group of EA is in contact with N111 (Figure 6d–f). Finally, analysing the interactions between clonidine and α_2C_-AR_6KUW_, it is noted that the 2-aminoimidazoline group creates two hydrogen bonds with D131, and then the dichlorophenyl ring is stabilized by two π-π interactions with F398 and Y402 (Figure 6g–i).

#### 3.1.2. MDs of EA Complexed with α_2_-ARs

For each complex, the best docked pose of EA, yohimbine and clonidine into α_2_-ARs binding pockets were chosen as the starting point for 50 ns of molecular dynamics simulations (MDs). MDs results were investigated in terms of stability of the complexes and conformational flexibility of α_2_-ARs in presence of the EA, with respect to the partial agonist and the antagonist, such as the clonidine and the yohimbine, respectively, by monitoring the single contributions of hydrophobic, water bridges and hydrogen bonding interactions.

**Figure 7:**
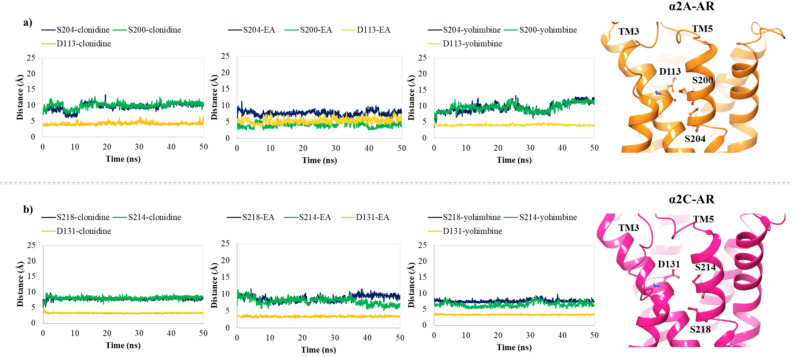
Plots of the distances between the centroid atom of EA, clonidine and yohimbine and (**a**) D113 (orange), S200 (green) and S204 (blue) into the α_2A_-AR structure; (**b**) D131 (orange), S214 (green), and S218 (blue) into the α_2C_-AR structure, throughout 50 ns of MDs. Right-hand images show zoomed-in context of the pivotal residues, depicted in orange and magenta carbon ball and sticks, involved in the α_2A_-AR and α_2C_-AR binding pockets, respectively.

In order to hypothesize the partial agonist or antagonist activity of EA, based on the importance of the interaction with aspartic acid on TM3 and the serine residues contained in TM5, as reported in previous studies [48], we monitored the distance between the centroid atom of EA, clonidine and yohimbine structures and the Cα of these critical residues, in α_2A_ (D113, S200, S204) and α_2C_ (D131, S214, S218) adrenoreceptors. Particularly, for the α_2A_-AR structure, a lower distance between EA and S200 was found with respect to that of clonidine and yohimbine (Figure 7a), with the average values equal to 7.3 Å, 10.0 Å and 10.8 Å, respectively. As already understood, S200 and S204 play a crucial role in the activation of aminergic GPCRs such as the α_2_-AR. The lack of flexibility of EA and its peculiar structure with hydrophobic core and hydrophilic ends allow it to reside in the TM5 area, which may be the cause of its partial agonism compared to the antagonist profile of yohimbine, which seems to move away from the TM5 in the last 10 ns of dynamics. Meanwhile, for the α_2C_-AR structure, all three investigated compounds remain stable in the orthosteric binding pocket (Figure 7b). No significant differences can be observed, except for EA which seems to be prone towards S214 at the end of the MDs.

Taking into account the dynamic behaviour of EA into the α_2A_-AR_6KUX_ structure with respect to the agonist and the antagonist, it is noticed that EA shifts towards R405 to form a π-cation interaction for 31% of MDs, losing the initial binding to D113. Its hydroxyl groups bind S90 and Y109 for 24% and 25% of MDs, respectively. Trajectories of clonidine and yohimbine present some similarities, such as their ability to interact with D113, Y394 and F412 residues, with an additional water bridge and H-bond with the E189 (36%) and I190 (95%) for the yohimbine in the complex with α_2A_-AR_6KUX_. Meanwhile, S200, which belongs to the TM5, stabilizes the clonidine into the α_2A_-AR_6KUX_ pocket by means of a water-bridge for 46% of MDs, underlining the key binding elements in common to the endogenous ligands and, consequently, its role as a partial agonist.

Curiously, even if EA is not able to maintain the interaction with D113, it is well stabilized during the whole trajectory in the complex with the α_2A_-AR_6KUY_ structure. Its conformational restraint provided by the hydrophobic core and the presence of the four hydroxyl groups results in a stronger binding affinity to drive EA within the α_2A_-AR_6KUY_ pocket for a good anchoring system than that of both the partial agonist and antagonist structures. Indeed, aromatic residues such as F116 and F390 form π-π interactions with the gallic acid motifs of EA for 38% and 98% of MDs. Moreover, S204, F408, F410 and F412 residues show a water-bridge interaction for around 30–60% of the trajectory. Regarding clonidine-α_2A_-AR_6KUY_ complex MDs, the 2-aminoimidazoline group binds D113 (30%) and F414 (33%) through an H-bond and a π-cation interaction, respectively. Additionally, the dichlorophenyl ring is oriented between Y394 and F391, engaging π-π interactions for a half run of MDs. Instead, analysing MDs of yohimbine-α_2A_-AR_6KUY_, its major flexibility allows maintenance of the interaction between the positively charged amine group in the pyrido[1,2-b]isoquinoline and D113 (38%) and L110 (53%) residues. This moiety also forms the successful anchoring π-π interaction with the phenylalanine at position 390 and 391 for 34% and 45% of MDs, respectively.

Conversely, all the three investigated compounds interact with similar but non identical subsets of residues in the α_2C_-AR structure. In particular, during the whole simulation, it can be observed that the 2-aminoimidazoline group of clonidine, the pyrido[1,2-b]isoquinoline of the yohimbine, and the two hydroxyl groups of one gallic acid motif of EA participate in the pivotal interaction with the side chain oxygens of D131 (Figure 5b), which is involved in adrenergic signalling for 96%, 99% and 99% of MDs, respectively. Y402 also provides a significant π-cation with clonidine (48%) and π-π interactions with both EA (40%) and yohimbine (42%). One gallic motif in EA displays two π-cation interactions with K420 for 55% and 31% of MDs. Carefully looking at yohimbine accommodation into the α_2C_-AR pocket during MDs, it appears that the indole moiety and the carboxyl group participate in major interactions with F398 (57%), and L204 (65%), respectively. Moreover, the hydroxyl group of yohimbine displays one water bridge with E112 (54%).

**Figure 8:**
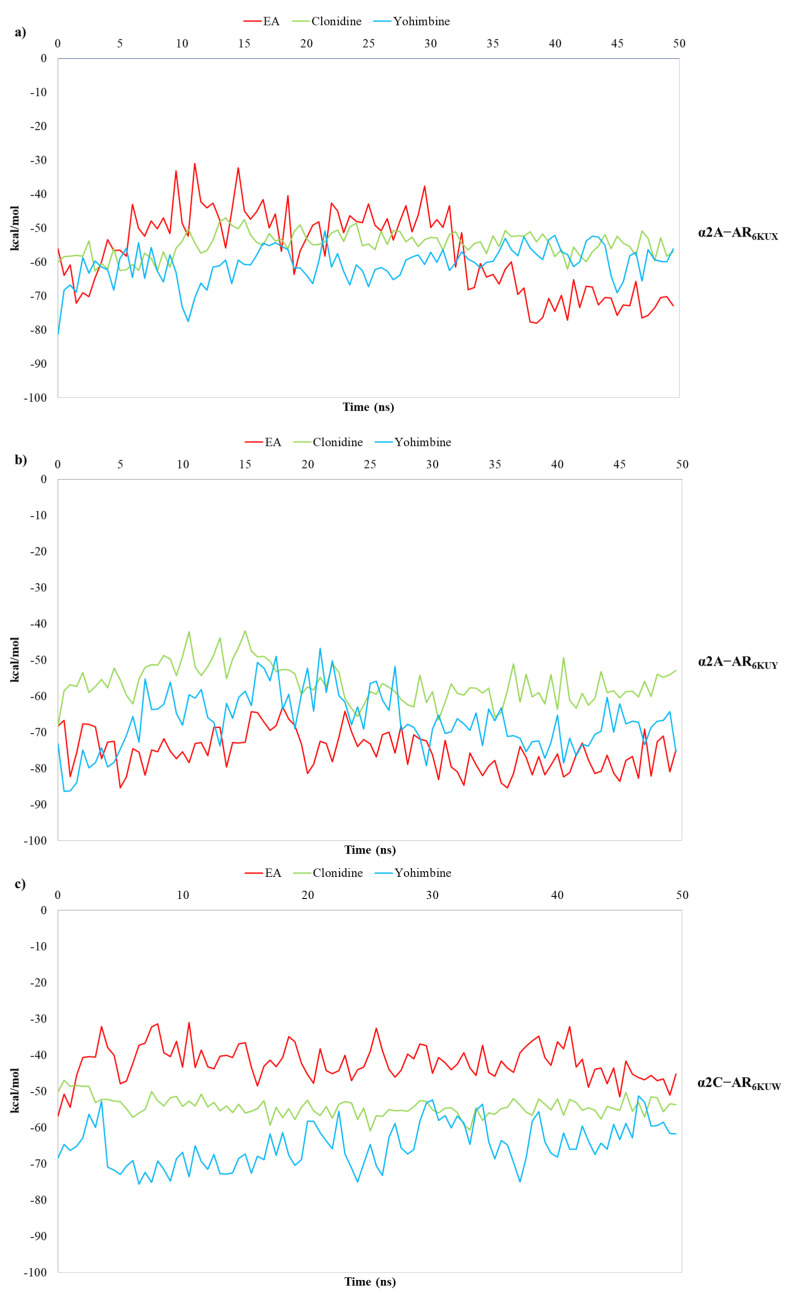
Plots of MM/GBSA trend for EA, clonidine and yohimbine in complex to (**a**) α_2A_-AR_6KUX_, (**b**) α_2A_-AR_6KUY_, and (**c**) α_2C_-AR_6KUW_, during 50 ns of MDs.

Finally, the binding free energies calculated by carrying out the MM/GBSA method, known to be one of the rigorous and efficient methods to predict relative binding affinities, has been useful for evaluating the strength of EA’s affinity over the α_2_-ARs binding pocket [49,50,51,52,53,54,55,56,57,58,59]. In this way, 1000 snapshots from 50 ns of MDs are extracted for the binding free energy calculations of both the α_2_-ARs-EA complex and the known partial agonist and antagonist in complex to α_2_-ARs [60,61]. The results of the calculated free energy trends for EA, in comparison with clonidine and yohimbine, are reported in Figure 8a–c.

MM/GBSA analysis reveals that the average calculated binding free energies (ΔG*_bind_*) of EA, clonidine and yohimbine complexed with α_2A_-AR_6KUX_ (Figure 7a) are −57.02, −54.86 and −60.69 kcal/mol, respectively, during the whole trajectories. Looking at the MM/GBSA trend, it can be seen that EA may behave like a partial agonist, strengthening its non-bonded interactions throughout MDs. Instead, the average values of −75.22, −56.41, −66.89 kcal/mol are calculated for EA, clonidine and yohimbine into α_2A_-AR_6KUY_, respectively, thus resulting in a more stabilizing effect of EA over the pocket of α_2A_-ARs (Figure 7b). In the α_2C_-AR_6KUW_, the MM/GBSA trend shows that yohimbine is associated to the higher binding free energies than that of both clonidine and EA, with average values equal to −64.88, −54.29 and −42.00 kcal/mol, respectively (Figure 8c).

### 3.2. Pharmacological In Vitro Functional Studies

#### 3.2.1. α_2__A_ and α_2C_ Receptor Proteins in Hippocampal Lysates

**Figure 9:**
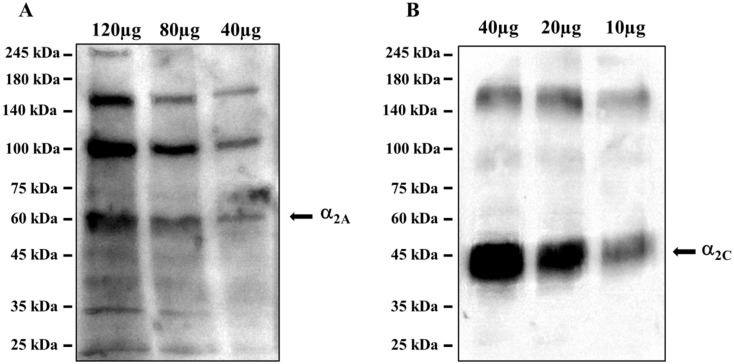
Mouse hippocampal lysates are endowed with (**A**) the α_2A_-AR and (**B**) the α_2C_-AR proteins. The images are representative of three analyses run on different preparations. Protein weights are expressed in kDa.

The ascending noradrenergic projections from the Locus coeruleus to the hippocampus possess in their preterminal varicosities and terminals presynaptic inhibitory release-regulating α_2_-ARs, as confirmed by the Western blot analysis of the mouse hippocampal lysates, which demonstrated the presence of the α_2A_-AR (Figure 9A) and the α_2C_-AR (Figure 9B) proteins, having a mass consistent with the monomeric structure (45 kDa and 50 kDa for the α_2A_ and α_2C_ receptor proteins, respectively), but also of polymeric associations, particularly evident in the case of α_2A_-AR. The analysis unveiled a direct correlation between the intensity of the immune-positivities and the protein content loaded in each lane. We did not investigate the presence of the α_2B_-AR protein because of its preferential peripheral expression [28].

These results confirmed the presence of α_2A_-AR and α_2C_-AR proteins in the hippocampus of adult mice, opening the possibility to focus on this brain region for in vitro functional studies to verify the intrinsic activity of EA on naïve α_2_-ARs.

#### 3.2.2. Ellagic Acid Mimics Clonidine at the Presynaptic Release-Regulating α_2_ Autoreceptors in Hippocampal Noradrenergic Nerve Endings: Antagonism by Yohimbine

To verify, by a functional point of view, if naive α_2_ARs could represent a specific cellular target of EA, we isolated synaptosomes from the hippocampus of adult male mice, and quantified in release experiments the impact of EA on the NA exocytosis. Experiments were carried out by using the technique of the “up-down superfusion of a thin layer of synaptosomes”, which is widely recognized as an approach of choice to prove, by a functional point of view, the existence of receptors in nerve endings, and to pharmacologically characterize the affinity and the intrinsic activity of ligand(s) acting at selected naïve receptor subtypes (see for experimental and technical details in the Method section, Figure 2).

**Figure 10:**
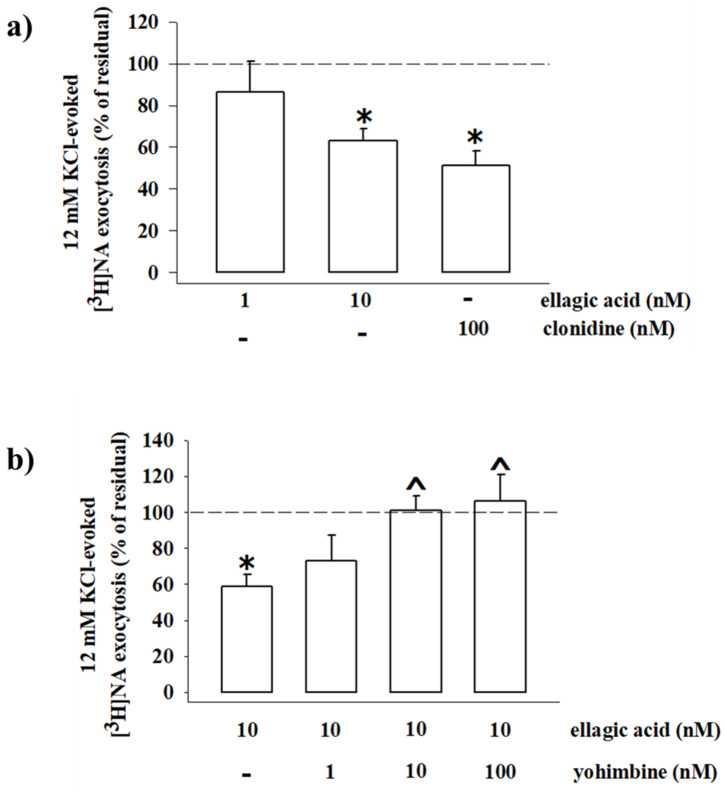
Ellagic acid mimics clonidine in inhibiting the ^3^[H]noradrenaline (^3^[H]NA) exocytosis from mouse hippocampal synaptosomes, and its effect is reversed by yohimbine. (**a**) Effects of ellagic acid (1 and 10 nM) and clonidine (100 nM) on the 12 mM KCl-evoked ^3^[H]NA release from mouse hippocampal synaptosomes. (**b**) Effect of ellagic acid (10 nM), alone or concomitantly added with yohimbine (1–100 nM), on the 12 mM KCl-evoked ^3^[H]NA release from mouse hippocampal synaptosomes. Results are expressed as percentage of residual of the 12 mM KCl-evoked tritium release. Data represent the means ± SEM of four to five experiments run in triplicate. * *p* < 0.05 versus 12 mM KCl-induced tritium overflow; ^ *p* < 0.05 versus 12 mM KCl/10 nM ellagic acid-induced tritium overflow.

Consistent with the inhibitory nature of the presynaptic α_2_-ARs, the exocytotic-like release of preloaded [^3^H]NA elicited by 12 mM KCl-enriched solution was significantly reduced (−48.80%, *n* = 4, *p* < 0.05, result expressed as % of inhibition) when the α_2_-ARs agonist clonidine (0.1 µM) was added concomitantly to the depolarizing stimulus. An almost comparable reduction of [^3^H]NA exocytosis was observed when synaptosomes were exposed to the KCl-enriched solution containing 10 nM EA (−36.80, *n* = 5, *p* < 0.05); a lower concentration (1 nM) of the natural compound slightly, although not significantly (−13.74, *n* = 3, n.s.), affected the tritium overflow (Figure 10a).

We asked whether the inhibitory effect elicited by EA involves the presynaptic release-regulating α_2_-ARs. To answer the question, yohimbine, a selective α_2_ autoreceptor antagonist, was added concomitantly to EA (10 nM) to compete with the natural compound for the binding at the presynaptic α_2_-AR. It is worth reminding that this drug, unable on its own to affect the tritium exocytosis, efficiently recovers the inhibitory effect elicited by clonidine on the 12 mM KCl-evoked tritium release (not shown but see [20,23,54,56,62,63,64]). Accordingly, yohimbine (1–100 nM) prevented, in a concentration-dependent fashion, the inhibitory effect elicited by 10 nM EA (Figure 10b).

On the whole, these functional observations demonstrate that EA activates naïve presynaptic release-regulating α_2_-ARs in hippocampal noradrenergic nerve endings. The natural compound mimics clonidine, behaving as an agonist at the naïve α_2_-ARs receptors. The agonist-like intrinsic activity of EA at these receptors is confirmed by the finding that the α_2_-AR antagonist yohimbine, devoid of intrinsic regulatory activity, prevents the agonist-like activity of EA at the naïve α_2_-ARs.

## 4. Conclusions

The peculiar structure of EA, characterized by a hydrophobic core and hydrophilic ends, led to its accommodation in the TM5 area, close to S200 and S204 which play a crucial role for activation of aminergic GPCRs, such as the α_2_-AR. Thermodynamic investigation revealed that EA is well stabilized into the α_2A_-AR binding site, if compared to the known partial agonist and antagonist. These structural findings highlight the promising role of EA as a partial agonist. Despite the lack of a positive region in the EA structure able to anchor the acid group of a conserved aspartate residue, this natural compound shows strong interaction in the binding pockets of α_2_-AR. Indeed, by MDs and MM/GBSA analyses, the hydrophobic residues surrounding the α_2A_-AR pocket confer specificity on the intermolecular interactions, and hence lead to favourable binding of EA in the α_2A_-AR, with respect to α_2C_-AR.

The in silico conclusions were verified in functional studies carried out with the mouse hippocampal synaptosomes bearing naïve α_2_-ARs. The experiments confirmed that EA has an intrinsic activity at the naïve α_2_-ARs. EA behaved at these receptors as an agonist, efficiently inhibiting NA exocytosis. The intrinsic agonist activity of the natural compound was definitively proved by the finding that the α_2_-AR antagonist yohimbine concentration-dependently prevented the EA-mediated inhibition of NA exocytosis. Unfortunately, the lack of drugs able to discriminate between the α_2A_-AR and the α_2C_-AR makes it impossible to characterize, by a functional point of view, the α_2_-AR subtype(s) involved in the EA-mediated control of NA exocytosis. Furthermore, the data so far available do not allow conclusion of whether EA acts as a partial or a full agonist at the presynaptic α_2_-ARs. Despite these limitations, the in vitro data confirms, by a functional point of view, that naive α_2_-ARs represent a specific site of action of EA. Taking into consideration the wide distribution of the α_2_-ARa, it seems conceivable to confirm that these receptors would mediate both peripheral and central EA-induced effects potentially involved in the nutraceutical activities of this natural compound.

## Figures and Tables

**Table 1:** Glide score, calculated in kcal/mol, of the best yohimbine, EA and clonidine docked pose towards α_2A_-AR_6KUX,_ α_2A_-AR_6KUY_ and α_2C_-AR_6KUW_ structures.

	α_2A_-AR_6KUX_	α_2A_-AR_6KUY_	α_2C_-AR_6KUW_
	Glide SP Score *	Glide SP Score *	Glide SP Score *
Yohimbine	−7.86	−7.62	−9.12
EA	−6.56	−7.43	−5.61
Clonidine	−4.85	−6.02	−4.39

* Glide SP score is calculated in kcal/mol.

## Data Availability

The data presented in this study are available in the article.
